# Knockdown of sexually differentiated vasopressin expression in the bed nucleus of the stria terminalis reduces social and sexual behaviour in male, but not female, mice

**DOI:** 10.1111/jne.13083

**Published:** 2022-01-02

**Authors:** Nicole Rigney, Adam Zbib, Geert J. de Vries, Aras Petrulis

**Affiliations:** ^1^ Center for Behavioral Neuroscience Neuroscience Institute Georgia State University Atlanta Georgia USA

**Keywords:** bed nucleus of the stria terminalis, mice, sex differences, social behaviour, vasopressin

## Abstract

The neuropeptide arginine‐vasopressin (AVP) has long been implicated in the regulation of social behaviour and communication, but the sources of AVP release relevant for behaviour have not been precisely determined. Ablations of the sexually dimorphic AVP cells within the bed nucleus of the stria terminalis (BNST), which are more numerous in males, affect social behaviour differently in males and females. However, it is unknown whether these behavioural effects are caused by a reduction of AVP or of other factors associated with these cells. To test the role of AVP specifically, we used an shRNA viral construct to knock down AVP gene expression within the BNST of wild‐type male and female mice, using scrambled sequence virus as a control, and evaluated subsequent changes in social behaviours (social investigation, ultrasonic vocalization (USV), scent marking, copulation, and aggression), or anxiety‐like behaviours (elevated plus maze). We observed that, in males, knockdown of AVP expression in the BNST strongly reduced investigation of novel males, aggressive signalling towards other males (tail rattling, USV), and copulatory behaviour, but did not alter attack initiation, other measures of social communication, or anxiety‐like behaviours. In females, however, BNST AVP knockdown did not alter any of these behaviours. These results point to differential involvement of AVP derived from the BNST in social behaviour.

## Graphical abstract

Several studies point at a sexually differentiated role of vasopressin (AVP) in social behaviour, but precisely which AVP sources are involved is not fully understood. BNST AVP knockdown in male, but not female, mice reduced social approach and communicative behaviors directed toward same‐sex conspecifics, without changing offensive attacks, anxiety‐related behaviors, and overall activity. BNST AVP knockdown also reduced male, but not female, sexual behaviour. AVP in the BNST contributes to sex differences in social behavior and its regulation.

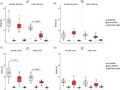

## 1 INTRODUCTION

The neuropeptide arginine‐vasopressin (AVP) has been strongly implicated in the regulation of social behaviour and communication across species.^1^, ^2^, ^3^, ^4^, ^5^ Many studies report that vasopressin, acting via V1a receptors (V1aR), modulate these behaviours in sexually different ways;^2^, ^6^, ^7^ however, the sources that drive these sex differences are not well described. In most animals, AVP is synthesized in several cell groups, each of which project to distinct brain areas.^8^, ^9^, ^10^ Two sources likely to contribute to sexually differentiated effects of AVP are the neurons in the bed nucleus of the stria terminalis (BNST) and medial amygdala (MeA), which contribute to the most pronounced sex differences in AVP innervation of the brain.^11^

Several studies suggest a sexually differentiated role for AVP cells in the BNST in social behaviour. For example, injections of AVP or AVP antagonists in BNST projection sites such as the lateral septum, lateral habenular nucleus, and ventral pallidum^10^ affect social behaviour differently in males and females in rats and mice.^12^, ^13^, ^14^, ^15^ In addition, partial knockdown of AVP gene expression in the BNST reduces male, but not female, social interactions, while increasing male, but not female, aggression in finches.^4^, ^16^, ^17^ In rats, intermale aggression correlates with AVP release in the septum and is reduced by intraseptal AVP antagonist application.^18^ Overall, these studies indicate that BNST AVP is important for male‐male interactions and for certain aspects of male prosocial communication, while playing a lesser role in female social behaviour and communication.

Recently, we directly tested whether cells expressing AVP in the BNST are involved in social behaviours in mice.^19^, ^20^ We found that ablations of these cells reduced male‐male investigation and increased male scent marking toward female stimuli.^19^ These studies, however, left the critical question unresolved as to whether AVP or other neuroactive substances are responsible for the effects seen after removal of BNST AVP cells. Consequently, in order to specifically target AVP, we reduced AVP‐expression in the BNST using viral expression of a short hairpin RNA (shRNA) targeted against AVP mRNA and tested the effects of this manipulation on social investigation, ultrasonic vocalization (USV), and urine marking, all aspects of mouse communication that show pronounced sex differences.^21^, ^22^, ^23^

## 2 METHODS

### 2.1 Animals and husbandry

All mice were maintained at 22°C on a 12:12 reverse light cycle with food and water available ad libitum and housed in individually ventilated cages (Animal Care Systems) with ALPHA‐dri bedding (Shepherd Specialty Papers, Watertown, TN), a nestlet square, and a housing tube. All animal procedures were performed in accordance with the Georgia State University Animal Care and Use Committee regulations and the National Institutes of Health Guide for the Care and Use of Laboratory Animals.

#### 2.1.1 Subjects

Forty‐eight male and female C57BL/6J mice between 8 and 12 weeks of age were obtained from Jackson Laboratories (stock # 000664) and were singly‐housed for a minimum of one week prior to testing.

#### 2.1.2 Stimulus animals

CD1(ICR) mice (Charles River Laboratories, Wilmington, MA, USA) were used as stimuli for behavioural testing and to provide male and female subjects with social experience, because strain differences between subjects and stimulus mice increase social investigation.^24^ Mice were used at 9–16 weeks of age and were novel to the subject to which they were exposed.

Female stimulus mice were grouped‐housed, ovariectomized, and implanted with an estradiol capsule (GDX+E; see below), and given two sexual experiences before testing. Two groups of stimulus males were used for behavioural testing. Mice that were used as subordinates in the home cage aggression tests and to provide aggressive experience to subjects, were grouped‐housed, gonadectomized (GDX), and subjected to two aggressive encounters with a dominant male (see below). Mice in the second group, which provided sexual experience to female subjects and served as sexual partners during copulatory tests as well as stimulus animals in the three‐chamber social test, were singly‐housed, gonadectomized, implanted with testosterone (GDX+T; see below), and then given two sexual experiences before testing.

### 2.2 shRNA virus

We used an adeno‐associated virus (AAV) expressing a short hairpin RNA (shRNA) to target *Avp* mRNA (AAV8‐GFP‐U6‐mAVP‐shRNA; titer: 1.0 × 10^13^ GC/ml; lot: 190930#33; sense target sequence: GGATGCTCAACACTACGCTCTCTCGAG AGAGCGTAGTGTTGAGCATCC; Vector Biolabs, Malvern, PA) and an AAV‐expressing a scramble shRNA (AAV8‐GFP‐U6‐scramble‐shRNA; titer: 4 × 10^13^ GC/ml; lot: 19044–190820; Vector Biolabs, Malvern, PA), which does not target any known sequence as a control. The *Avp*‐shRNA target sequence demonstrated 91% knockdown in vitro as tested by the manufacturer. BLAST searches did not reveal significant target alignment between the *Avp*‐shRNA sequence and other coding mRNAs, including oxytocin, indicating that the shRNA targeted *Avp* mRNA specifically. Both scramble (control) and *Avp*‐shRNA vectors express green fluorescent protein (GFP) to allow for visualization of the infected neurons.

### 2.3 Surgery

All surgeries were carried out using 1.5%–2.5% isoflurane gas anesthesia in 100% oxygen; 3 mg/kg of carprofen was given subcutaneously before surgery to reduce pain.

#### 2.3.1 Stereotaxic surgery

Mice were positioned in a stereotaxic frame (David Kopf Instruments) with ear and incisor bars holding bregma and lambda level. After a midline scalp incision, a hand‐operated drill was used to make holes in the skull, exposing the dura. Then, 200 nl of *Avp*‐shRNA or scramble‐sequence virus was delivered bilaterally to the BNST (coordinates: AP + 0.15 mm; ML ± 0.8 mm; DV 4.4 mm) at a rate of 100 nl/min using a 5 μl Hamilton syringe with a 30‐gauge bevelled needle mounted on a stereotaxic injector. Following virus delivery, the syringe was left in place for 10 min before slowly withdrawing it from the brain.

#### 2.3.2 Gonadectomy and hormone treatment

Testes were removed after cauterizing the ductus deferens and blood supply via a midline abdominal incision. Silastic capsules (1.5 cm active length; 1.02 mm inner diameter, 2.16 mm outer diameter; Dow Corning Corporation) were filled with crystalline testosterone (T; Sigma, St. Louis, MO, USA) and inserted subcutaneously between the scapulae after gonadectomy; this procedure leads to physiological levels of T.^25^, ^26^ To further reduce aggression in stimulus animals,^27^ some males were gonadectomized, but did not receive a T implant (GDX).

The ovaries of stimulus female mice were removed after cauterizing its blood supply via an abdominal incision at the uterine horn. Silastic capsules (0.7 cm active length; 1.02 mm inner diameter, 2.16 mm outer diameter; Dow Corning Corporation, Midland, MI, USA) containing estradiol benzoate (E; diluted 1:1 with cholesterol) were implanted subcutaneously in the scapular region immediately following ovariectomy (GDX+E).^28^, ^29^ To induce sexual receptivity, stimulus females were injected subcutaneously with 0.1 ml of progesterone (500 μg dissolved in sesame oil, Sigma, St. Louis, MO, USA) 4 h preceding sexual experience, urine collection, and behavioural testing.^30^

### 2.4 Urine collection

Pooled urine samples were collected from stimulus females induced into estrus and from stimulus males (5–10 mice per sample). Estrous state was verified by color, swelling, and expanded size of the vaginal opening.^31^ To collect urine, mice were picked up by the tail base and held by dorsal neck skin; this method typically induced urination. If the mouse did not urinate, stroking its belly from an anterior to posterior direction stimulated bladder voiding. Each mouse provided 40–80 μl of urine that was pooled into a 1.5 ml Eppendorf tube. Urine samples were used fresh within 1 h of collection to prevent chemosignal degradation.^32^

### 2.5 Social experience

As opposite‐sex sexual experience and attaining competitive status (“social dominance”) promote communicative behaviours,^32^, ^33^ mice received social experience over five consecutive days (sexual encounters on days 1 and 4, aggressive encounters on days 2 and 5; no encounters on day 3).

#### 2.5.1 Sexual experience

Subjects were given two opportunities to interact with either a stimulus female (for male subjects) or a stimulus male (for female subjects). A sexually‐experienced stimulus mouse was placed in the subject's home cage and removed the next day (first experience) or after 90 min (second experience). Subjects that did not ejaculate or elicit ejaculation (females) during the second sexual experience were removed from further testing.

#### 2.5.2 Aggressive experience

Male subjects were exposed to two interactions with a subordinate stimulus male treated with 50 μl of GDX+T male urine applied to its back, a manipulation which elicits offensive aggression in subjects.^27^, ^34^, ^35^ Stimulus males were placed in the subject's home cage and removed after the subject's first offensive attack (biting) within a 10‐min period. All subject males attacked the intruder stimulus male, and all stimulus males displayed submissive behaviour, defined as defensive postures (e.g., on‐back postures, fleeing, and nonsocial exploring^36^). Female subjects were exposed to a female intruder; however, this did not elicit attacks from either animal.

### 2.6 Experimental procedure

All testing occurred within the first 5 h of the dark cycle under red light illumination (27 lux), with the exception of the elevated plus maze (EPM), which took place in bright illumination (435 lux). All tests were scored by an experimenter blind to the genotype of the subject and testing occurred across multiple cohorts of subjects. Three weeks after viral injections, subjects were habituated to the testing room and apparatus by handling and placing mice for 5 min in the three‐chamber apparatus (see below) each day for three days. On experimental days, subjects were adapted to the experimental room for 15 min prior to testing. First, we tested mice on an EPM to test for anxiety‐related behaviour.^37^ Mice were then tested in the three‐chamber apparatus over six days with a one day break on the fourth day. Lastly, copulatory and aggressive behaviour were measured sequentially, with a day in between, in the subject's home cage. Female subjects were tested irrespective of oestrous cycle day, except during copulation testing, when they were in behavioural estrus. Prior research indicates minimal effects of oestrous cycle on female mouse communicative behaviour,^38^, ^39^, ^40^ nevertheless the oestrus cycle was monitored throughout the study. Following testing, subjects were sacrificed and their brain tissue was processed using immunohistochemistry to detect AVP, oxytocin (OT), galanin (GAL), and GFP‐immunoreactive (‐ir) cells and fibres in the BNST, paraventricular nucleus of the hypothalamus (PVN), and lateral septum (LS).

### 2.7 Social behaviour

USV, urine marking, and social investigation were recorded in an acrylic three‐chamber apparatus (Ugo Basile, Gemonio (VA) Italy; dimensions: 60 × 40 × 22 cm).^41^, ^42^, ^43^ Instead of a solid floor, the apparatus was placed on absorbent paper (Nalgene Versi‐dry paper, Thermo Fisher Scientific) to allow for accurate measurement of urine marking. One corner of the apparatus had a cylindrical cage (8 cm (D), 18 cm (H); 3 mm diameter steel bars, 7.4 mm spacing) that housed a stimulus animal, the opposite corner had a similar cage that was left empty. For testing with social odors, subjects had access to 50 μl of fresh urine from a stimulus animal or 50 μl saline pipetted onto a clean piece of filter paper (3 cm^2^) that was taped on the outside of cages. The location of stimulus and the “clean” cage were counterbalanced across animals. After placing the subject in the center of the middle chamber, we measured, across a 5‐min trial, close investigation of clean and stimulus cages as well as USV and urine marking, as described below. After testing, the apparatus and cages were thoroughly cleaned with 70% ethanol and allowed to dry before further testing. In all cases, male or female urine stimulus was presented first (day one), followed by exposure to a stimulus animal of the same sex the following day (day two); this order was then repeated one day later for the opposite sex. In this fashion, mice first experienced a weak stimulus (urine), then a stronger social stimulus (live animal). The order of male and female stimuli presentation was counterbalanced across subjects.

#### 2.7.1 Investigation and ultrasonic vocalizations

Close investigation was defined as time spent sniffing within 2 cm of the stimulus or clean cage; climbing on the cage was not scored as investigation. USVs were detected using a condenser microphone connected to an amplifier (UltraSoundGate CM16/CMPA, 10–200 kHz, frequency range) placed 4 cm inside the apparatus and directly above the center compartment. USVs were sampled at 200 kHz (16‐bit) with target frequency set to 70 kHz (UltraSoundGate 116Hb, Avisoft Bioacoustics, Berlin, Germany). Recordings were then analyzed using a MATLAB (MATLAB, Mathworks, RRID:SCR_001622) plug‐in that automates USV analysis.^44^ Using this program, sonograms were generated by calculating the power spectrum on Hamming‐windowed data and then transformed into compact acoustic feature representations (Gammatone Filterbank). Each 200‐ms window containing the maximum USV syllable duration was then clustered via machine learning algorithms into USV syllable types (repertoire units) based on time‐frequency USV shape. Repertoire units that appeared as background noise were discarded. We collapsed and counted across all syllable types and analysed the total number USVs produced by each subject.

#### 2.7.2 Urine marking

Following testing, the substrate sheet was allowed to dry for 1 h and then sprayed with ninhydrin fixative (LC‐NIN‐16; Tritech Forensics Inc.) to visualize urine marks.^22^, ^45^ After 24 h, sheets were imaged (Sony DSC‐S700 camera), binarized and analyzed using computer‐aided imaging software (ImageJ, RRID:SCR_003070). Urine marking was measured as the total area (pixels) of visualized ninhydrin urine marks in the entire arena.

#### 2.7.3 Copulatory and aggressive behaviour

To measure copulatory behaviour, a stimulus mouse was placed in the subject's home cage and then removed after 90 min had elapsed. The number of mounts, intromissions, and ejaculations and their latencies were recorded, along with mount rejections (female kicking male off during mounting attempt) by female subjects. To measure territorial aggression, subordinate stimulus males were placed in the subject's home cage and then removed after the subject's first offensive attack (biting and rolling) within a 10‐min period; the latency to first bite and to first rolling‐attack was recorded.

### 2.8 Elevated plus maze

The elevated plus maze (EPM) consisted of two open arms (30 × 5 × 0 cm) and two closed arms (30 × 5 × 25 cm) crossed perpendicularly and raised 60 cm above the floor. Subjects were placed at the arm intersection facing the open arm and were habituated to the apparatus for 1 min; subjects were then observed for an additional 5 min. Time spent in open and closed arms and the number of risk assessment behaviours (stretch‐attend posture, head‐dips) were manually scored from video.^46^ Subjects were removed from EPM data analysis if they fell off the EPM during testing.

### 2.9 Histology and immunohistochemistry

Mice were transcardially perfused with 50 ml of 0.1 M PBS (pH 7.4), followed by 50 ml of 4% paraformaldehyde in phosphate buffer (pH 7.2). Brains were immediately removed and post‐fixed in 4% paraformaldehyde overnight (4 °C) and then cryoprotected for 48 h in 30% sucrose. Coronal sections (30μm) of brain tissue were sectioned on a cryostat (Leica CM3050 S, Leica Biosystems) into four series and stored in cryoprotectant until immunohistochemical processing. Tissue from all subjects (*n* = 48) was stained for GFP, and AVP, whereas sections from a subset of subjects were stained for GAL (*n* = 10) or OT (*n* = 8).

Sections were removed from cryoprotectant and rinsed thoroughly in PBS. For optimal AVP/OT/GAL staining, we used an antigen retrieval step in which sections were incubated in 0.05 M sodium citrate in PBS at 70°C for 30 min and allowed to cool for 10 min prior to primary antibody incubation. To reduce endogenous peroxidase activity, tissue sections were incubated in 0.5% hydrogen peroxide in PBS for 15 min, followed by a blocking step for 1 h (10% normal goat or donkey serum). Sections were incubated in the appropriate primary antibody to AVP (1:50,000, guinea pig polyclonal, lot: A17901), OT (1:100,000, guinea pig polyclonal, lot: A17698), or GAL (1:40,000, rabbit polyclonal, lot: A17602) (Peninsula Laboratories International, Inc.) in 0.4% Triton‐X 100 for 24 h at room temperature. After incubation in primary antibody, sections were rinsed in PBS and then incubated for 1 h in goat antiguinea pig (lot: 151056) or goat anti‐rabbit (lot: 151508) biotinylated secondary antibody (1:600 Jackson ImmunoResearch, West Grove, PA, USA) in PBS with 0.4% Triton‐X 100. Sections were rinsed again in PBS and then incubated for 1 h in avidin–biotin complex (18 μl each of A and B reagents/ml PBS with 0.4% Triton‐X 100, ABC Elite Kit, Vector Laboratories, Burlingame, CA, USA). After rinsing in PBS and then in 0.175 M sodium acetate, sections were incubated in 3,3′‐diaminobenzidine HCl (0.2 mg/ml, Sigma) and hydrogen peroxide (1 μl/ml, Sigma) in a nickel‐sulfate solution (25 mg/ml, Sigma) for 15 min. The reaction was stopped by rinsing sections in sodium acetate. Stained tissue sections were mounted onto subbed glass slides and allowed to air‐dry overnight. Slides were then dehydrated in alcohols, cleared in xylenes, and coverslipped using Permount (Fisher Scientific).

For GFP staining, sections were removed from cryoprotectant and rinsed thoroughly in PBS. After several rinses, sections were incubated in primary antibody against GFP (1:5000, chicken polyclonal, lot: ab13970, Abcam) in 0.4% Triton‐X 100 for 24 h at room temperature. After incubation in primary antibody, sections were rinsed in PBS and then incubated for 2 h in goat anti‐chicken fluorescent (Alexa Fluor 488) secondary antibody (1:600, lot: ab150169, Abcam). Stained tissue sections were mounted onto subbed glass slides and coverslipped using Prolong Gold (Fisher Scientific).

### 2.10 Tissue analysis

**FIGURE 1:**
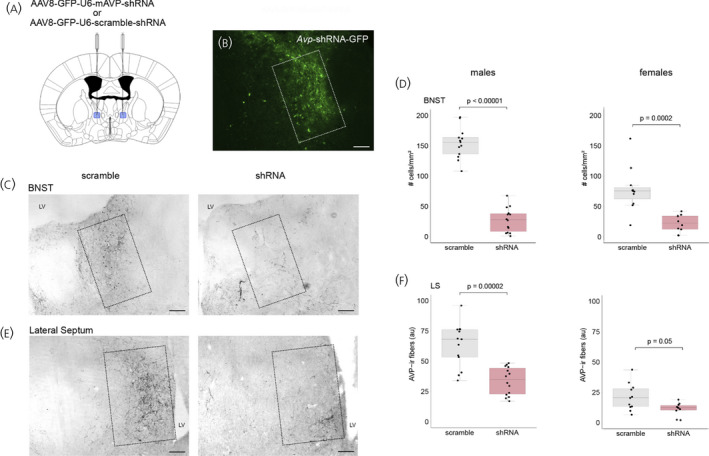
Histology. (A) Adenoassociated virus (AAV) expressing a short hairpin RNA (shRNA) to target *Avp* mRNA (AAV8‐GFP‐U6‐mAVP‐shRNA) or a control AAV expressing a scramble shRNA sequence (AAV8‐GFP‐U6‐scramble‐shRNA) and location of bilateral injection site in the bed nucleus of the stria terminalis (BNST). (B) Example image showing AAV‐GFP‐labelled cells in the BNST. (C) Example images of AVP‐immunoreactive (ir) cells within the BNST following a scramble shRNA AAV (left) or *Avp*‐shRNA AAV injections (right). Rectangles indicate position of AVP‐ir cells (D) Boxplots showing that AVP‐ir cell number is significantly lower in AVP shRNA‐injected male (*n* = 14, *p* < 0.00001) and female (*n* = 10, *p* = 0.0002) subjects compared to scramble shRNA‐injected controls (males: *n* = 13; females *n* = 11). (E) Example images of AVP‐immunoreactive (ir) fibres within the LS following scramble shRNA AAV (left) or *Avp*‐shRNA AAV (right) injections into the BNST. Images were taken at 10x magnification from sections from the same scramble shRNA or *Avp*‐shRNA AAV‐injected male subjects. Rectangles indicate position of AVP‐ir fibres. Scale bar = 50 µm (F) Boxplots showing that AVP‐ir fibre density is significantly lower in *Avp*‐shRNA‐injected male (*p* = 0.00002) and female (*p* = .05) subjects compared to scramble shRNA‐injected controls. Boxplots indicate individual data points, median, first, and third quartiles

Bilateral BNST images were taken at 10× magnification using a Zeiss Axio Imager M2 microscope (Carl Zeiss Microimaging), which transferred fluorescent images (FITC contrast reflector) to image analysis software (Stereo Investigator, MicroBrightField, RRID:SCR_002526). Imaging domains (2 mm^2^) were placed with reference to anatomic landmarks (ventricles, fibre tracts; Paxinos and Franklin, 2012). Only subjects with fluorescent labelled GFP cells limited to the BNST were included in the analysis (Figure 1A); subjects with viral spread to other regions (e.g., PVN) or with only unilateral infection were removed from analysis. AVP‐, OT‐, and GAL‐expressing cells in the BNST, AVP‐expressing cells in the PVN, and AVP fibre density in the LS, a prominent target for sex‐different BNST AVP‐expressing cells,^10^, ^47^ were measured and averaged across both hemispheres and over three sections (BNST and LS) or four sections (PVN). Subjects with <50% AVP reduction in the BNST were removed from analysis. Density of AVP‐ir fibres in the LS was measured by gray‐level thresholding of digitally captured images using ImageJ (NIH, http://imagej.nih.gov/ij). Background measurements were taken from an adjacent area with no AVP label and averaged across all brains. Specific AVP‐ir fibre density was calculated by subtracting the average nonspecific background from the AVP‐ir density measurement (pixels).

### 2.11 DeepLabCut and SimBA analysis

Deeplabcut (DLC) 2.2b8 was used to track eight body points (nose, ears, centre, left flank, right flank, tail base, and tail end) on the subject mice. A tracking dataset was created by labelling 20 frames from 10 randomly chosen male and female subject videos for a total of 200 labelled frames. To adequately train a network that could track mice movements, the generated dataset underwent 200,000 iterations of analysis under the default neural network, resnet_50.^48^ The resulting network was then applied to each subject's 305‐s video of their time spent in the 3‐chamber apparatus. Tracking point coordinates (CSV file) were produced for each video analysed and used for further analysis.

CSV files generated from DLC analysis and their corresponding videos were further analysed with Simple Behavioural Analysis (SimBA 0.84.1),^49^ an open source program that enables the automated classification of complex social behaviours. First, pixel/millimeter ratios were established for each video, and a region of interest analysis was used to calculate features related to distance, directionality, and time spent in each chamber of the 3‐chamber apparatus. The center body marker was the point of interest used to track subjects’ distance covered in the 3‐chamber apparatus to detect if BNST‐AVP knockdown influenced locomotor activity. Second, three rectangular regions of interest, each one outlining a different chamber in the apparatus, were generated to calculate the subject's time spent in each chamber. Lastly, a circular region of interest was outlined around the stimulus cage to measure head orientation, and therefore attention towards a distal stimulus, similar to social vigilance measurements that indicate increased anxiety in stressed animals.^50^ We measured this orientation behaviour when the subject's nose was oriented toward the centre of the circular region of interest and was a distance of 9–30 cm away from the centre of the circular region of interest.

### 2.12 Statistical analysis

All data were analysed and graphed in R (3.4.4; R Core Team, 2017). Data on histology, social investigation, movement, head orientation, time spent in chambers, urine marking, copulatory behaviour, and EPM met the assumptions of parametric statistical tests. Therefore, we analysed these data using a mixed‐model ANOVA with treatment (injections with *Avp*‐shRNA, scramble control) and sex (male, female) as between‐subject factors, and sex of stimulus (male, female) and stimulus location (stimulus cage, empty cage) as within‐subject factors when appropriate; these were followed by planned *t*‐tests comparing the treatment effects. For copulatory behaviour, which was measured for males and females differently, we used *t*‐tests. Differences in proportion of animals engaging in copulatory behaviours across treatments was assessed using a chi‐square (χ^2^) test. Measures of USVs and aggressive behaviour were not normally distributed and could not be transformed. Therefore, we analysed treatment effects on these behaviours using Mann‐Whitney U tests. Power analyses for each sample size were above 0.7 and post‐hoc comparisons report Bonferroni‐corrected *p*‐values. Exact *p*‐values are reported, except when *p* exceeds a significance threshold of 0.00001. Eta‐squared (η^2^), Cohen's D (d), and Phi (φ) are reported for standardized effect sizes.

## 3 RESULTS

### 3.1 shRNA effectively reduced AVP expression in BNST and LS

Males had more AVP‐immunoreactive (‐ir) cells in the BNST than females (*F*(1,45) = 30.34, *p* < 0.00001, η^2^ = 0.41). AVP knockdown caused an overall reduction in the number of AVP‐ir cells (*F*(1,45) = 154.46, *p* < 0.00001, η^2^ = 0.8). There was an interaction between treatment and sex (*F*(1,45) = 23.01, *p* = 0.00002, η^2^ = 0.3), which may be related to the knockdown reducing the number of AVP‐ir cells more in males than in females (82% in males (t(25) = 14.41, *p* < 0.00001, d = 5.8) and 72% in females (t(19) = 4.56, *p* = 0.0002, d = 2.1)) (Figure 1C,D).

Males had a higher density of AVP‐ir fibres in the LS than females (*F*(1,45) = 64.2, *p* < 0.00001, η^2^ = 0.61). AVP knockdown caused an overall reduction in the density of AVP‐ir fibres (*F*(1,45) = 24.74, *p* = 0.00002, η^2^ = 0.38). There was an interaction between treatment and sex *F*(1,45) = 5.29, *p* = 0.027, η^2^ = 0.11), with the reduction being larger in males than in females (Figure 1E,F).

The number of AVP‐ir cells was not altered in the PVN of male or female subjects (*F*(1,45) = 0.5, *p* = .47, η^2^ = 0.01), nor was there a reduction in the number of GAL‐ir (*F*(1,8) = 0.51, *p* = 0.5, η^2^ = 0.05) or OT‐ir cells (*F*(1,6) = 0.04, *p* = 0.85, η^2^ = 0.006) in the BNST in a subset of male subjects, indicating that the shRNA targeted *Avp* mRNA specifically and did not have off‐target effects (Figure S1).

### 3.2 BNST AVP knockdown reduced social investigation of novel males in males but not in females

**FIGURE 2:**
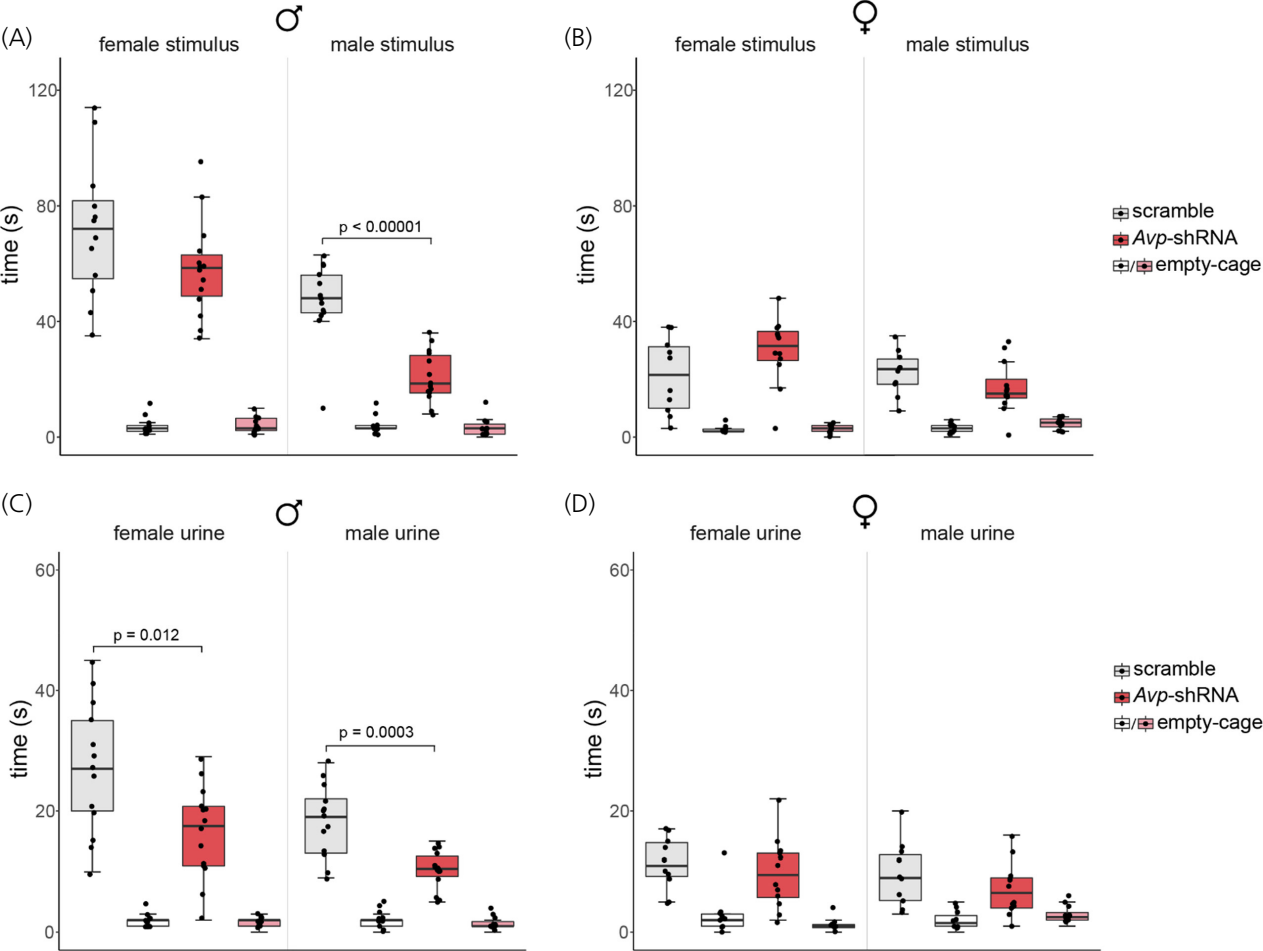
Social investigation. Boxplots indicate individual data points, median, first and third quartiles for time spent investigating wire cages containing male or female stimulus animals, or an empty wire cage within the three‐chamber apparatus. BNST AVP knockdown in males (A), but not females (B), decreased investigation of male (*p* < 0.00001) stimuli compared to controls. BNST AVP knockdown in males (C), but not females (D), decreased investigation of male urine (*p* = 0.012) and female urine (*p* = 0.0003) compared to controls

All subjects investigated cages with stimulus animals more than the empty cages (*F*(1,45) = 30.05, *p* < 0.00001, η^2^ = 0.6). Overall, AVP knockdown decreased the time animals investigated cages with stimulus animals (*F*(1,45) = 8, *p* = 0.007, η^2^ = 0.3). However, effects were limited to male animals, causing an interaction between sex and treatment (*F*(1,45) = 10.57, *p* = 0.002, η^2^ = 0.2). While control males investigated male stimuli more than did control females (male stimuli: t(21) = 5.2, *p* = 0.0001, d = 2.27; female stimuli: t(21) = 4.22, *p* = 0.001, d = 1.8), BNST AVP knockdown specifically reduced the time males spent investigating cages with stimulus males (t(25) = 6.01, *p* < 0.00001, d = 2.43). This eliminated a sex difference in the time that control males and females spent investigating male stimuli (t(21) = 1.06, *p* = 0.3, d = 0.2). AVP knockdown did not affect male subjects’ investigation of female stimulus animals (t(25) = 1.9, *p* = 0.14, d = 0.7) nor did it affect female subjects’ investigation of male (t(20) = 1.45, *p* = 0.32, d = 0.6) or female (t(20) = 1.66, *p* = 0.23, d = 0.7) stimulus animals (Figure 2A, B).

All subjects investigated cages with urine samples more than the empty cages (*F*(1,45) = 19.67, *p* = 0.00006, η^2^ = 0.4). Overall, AVP knockdown decreased the time animals investigated cages with urine samples (*F*(1,45) = 14.42, *p* = 0.0004, η^2^ = 0.3). However, as with stimulus animals, AVP knockdown effects were limited to male animals, causing an interaction between sex and treatment (*F*(1,45) = 6.08, *p* = 0.018, η^2^ = 0.18). While control males investigated urine more than did control females (male urine: t(21) = 3.7, *p* = .002, d = 1.6; female urine: t(21) = 4.3, *p* = 0.0006, d = 1.8), BNST AVP knockdown specifically reduced the time males spent investigating both male and female urine (male urine: t(25) = 4.42, *p* = 0.0003, d = 1.77; female urine: t(25) = 2.99, *p* = 0.012, d = 1.2). This eliminated a sex difference in the time that control males and females spent investigating male urine (t(21) = 2.1, *p* = 0.094, d = 0.6) but not for investigating female urine (t(21) = 2.5, *p* = 0.04, d = 0.8). AVP knockdown did not affect female subjects’ investigation of male (t(20) = 1.13, *p* = 0.27, d = 0.4) or female (t(20) = 0.75, *p* = 0.46, d = 0.3) urine (Figure 2C, D).

DeepLabCut and SimBA analysis revealed that subjects differed in time spent in the chamber containing stimulus animals (*F*(2,82) = 0.5, *p* = 0.44, η^2^ = 0.007). Overall, AVP knockdown increased the time animals spent in the chamber furthest away from the stimulus animals (*F*(2,82) = 4.35, *p* = 0.016, η^2^ = 0.09). There was no interaction between sex, treatment, and chamber location (*F*(2,82) = 1.3, *p* = 0.27, η^2^ = 0.02). However, post hoc analysis revealed that BNST AVP knockdown specifically increased the time males spent in the furthest chamber of the apparatus with stimulus males (male stimuli: t(25) = 2.78, *p* = 0.01, d = 1.1; female stimuli: t(25) = 0.9, *p* = 0.37, d = 0.3). There was also a trend toward BNST AVP knockdown decreasing the time males spent in the chamber with the stimulus male compared to controls (male stimuli: t(25) = 2.0, *p* = 0.056, d = 0.8; female stimuli: t(25) = 0.4, *p* = 0.73, d = 0.1). AVP knockdown did not affect female subject's time spent in the clean chambers (male stimuli: t(20) = 1.7, *p* = 0.11, d = 0.7; female stimuli: t(20) = 0.03, *p* = 0.98, d = 0.01) or the chamber with the stimulus animals compared to controls (male stimuli: t(20) = 1.3, *p* = 0.18, d = 0.6; female stimuli: t(20) = 0.9, *p* = 0.36, d = 0.3; Figure S2).

BNST AVP knockdown did not affect overall locomotion within the 3‐chamber apparatus ((*F*(1,45) = 1.55, *p* = .22, η^2^ = 0.018, Figure S3), and there was no interaction between treatment and sex (*F*(1,45) = 0.04, *p* = .85, η^2^ = 0.001). Additionally, BNST AVP knockdown did not affect the amount of time subjects spent oriented toward the stimulus cages at a 9–30 cm distance (Figure S4).

### 3.3 BNST AVP knockdown reduced male‐male USVs, but did not alter urine marking

**FIGURE 3:**
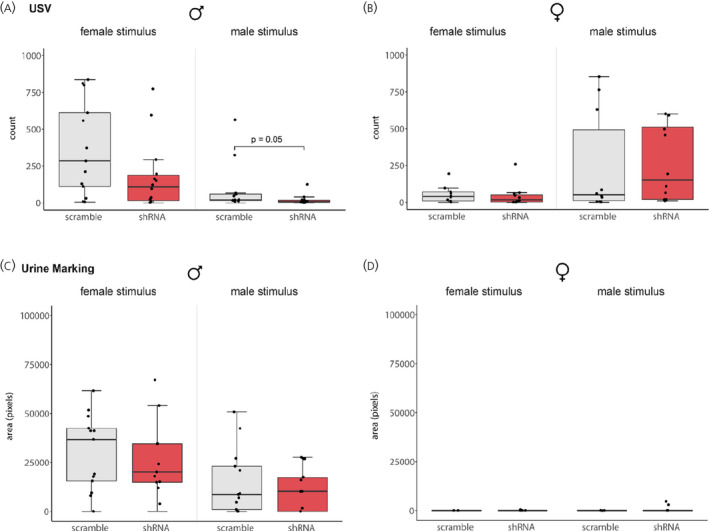
Ultrasonic vocalizations (USV) and urine marking within the three‐chamber apparatus. (A) BNST AVP knockdown reduced USVs produced during male‐male conditions (*p* = 0.05). (B) *Avp*‐shRNA and scramble shRNA‐ injected females did not differ in USVs produced during three‐chamber testing. (C, D) *Avp*‐shRNA and scramble shRNA‐injected males (C) and females (D) did not differ in urine marking (area covered) toward male or female stimuli during three‐chamber testing. Boxplots indicate individual data points, median, first and third quartiles

Most vocalizations were produced during male‐female interactions and least during female‐female interactions (Figure 3A,B). BNST AVP knockdown reduced overall USVs produced during social interactions (U = 234, *p* = 0.024, d = 0.55), However, this effect seems to be driven primarily by a reduction of USVs produced during male‐male interactions (male subjects with male stimuli: U = 45.5, *p* = 0.05, d = 0.68; male subjects with female stimuli: U = 57, *p* = 0.11, d = 0.6). USVs produced during interactions of female subjects and stimulus animals were much lower and unaltered following BNST AVP knockdown (female subjects and female stimuli: U = 45, *p* = 0.35, d = 0.1; female subjects with male stimuli: U = 67, *p* = 0.67, d = 0.04) (Figure 3A,B). AVP knockdown did not alter USVs produced during exposure to female urine (males: U = 77, *p* = 0.52, d = 0.1; females: U = 34, *p* = 0.09, d = 0.2) or male urine (males: U = 47, *p* = 0.42, d = 0.09; females: U = 77, *p* = 0.52, d = 0.2) (Figure S5).

Overall, males, but not females, produced urine marks (Figure 3C,D). Males produced more urine marks and covered a larger area with urine in the presence of a female than of a male stimulus (number of marks: *F*(1,25) = 16.53, *p* = 0.0004, η^2^ = 0.1; area: (*F*(1,25) = 29.713, *p* = 0.00001, η^2^ = 0.19)). BNST AVP knockdown did not affect the number of urine marks nor the area covered with those marks in the presence of stimulus animals (number of marks: *F*(1,25) = 0.4, *p* = 0.56, η^2^ = 0.001; area: (*F*(1,25) = 0.1, *p* = 0.76, η^2^ = 0.01))(Figure 3C,D) or urine samples (*F*(1,25) = 0.5, *p* = 0.51, η^2^ = 0.02)(Figure S5).

### 3.4 BNST AVP knockdown reduced offensive signalling, but not offensive attacks in males

**FIGURE 4:**
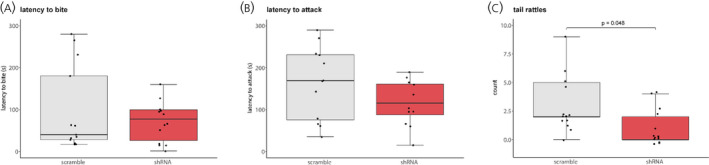
Aggressive behaviour. In males, BNST AVP knockdown did not alter the latency to bite (A) or latency to rolling attack (B) but did reduce the number of tail rattles during encounters with male intruders (C), *p* = 0.048. Boxplots indicate individual data points, median, first and third quartiles

Male subjects were the only ones to engage in aggressive attacks and tail rattling during the aggression tests. BNST AVP knockdown reduced tail rattles of subjects toward male intruders (U = 50, *p* = 0.048, d = 0.83); however, the latency to bite (U = 89, *p* = 0.9, d = 0.1) and attack (U = 73.5, *p* = 0.4, d = 0.1) the male intruder was similar between control and shRNA‐injected males (Figure 4A–C).

### 3.5 BNST AVP knockdown reduced copulatory behaviour in males

**FIGURE 5:**
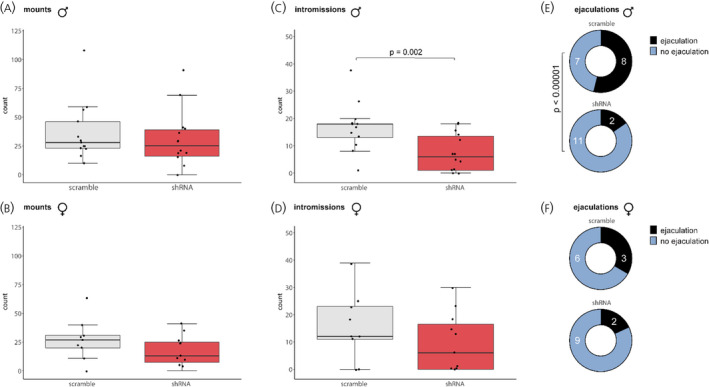
Copulatory behaviour. *Avp*‐shRNA and scramble shRNA‐ injected males (A) and females (B) did not differ in number of mounts performed (males) and number of times mounted (females). (C) *Avp*‐shRNA reduced the number of intromissions performed by males compared to controls (*p* = 0.002). (D) The number of intromissions received by females was unaltered. Pie chart summarizing the proportion of male subjects that ejaculated (E) and the proportion of male stimulus animals that ejaculated with female subjects (F). BNST AVP knockdown resulted in fewer males ejaculating (*p* < 0.00001). Boxplots indicate individual data points, median, first and third quartiles

In males, BNST AVP knockdown reduced the number of intromissions (t(25) = 3.13, *p* = 0.002, d = 0.9) but did not affect the number of mounts (t(25) = 0.7, *p* = 0.49, d = 0.2). Similarly, BNST AVP knockdown increased the latency to intromit (t(25) = 2.6, *p* = 0.002, d = 0.9), but did not affect the latency to mount (t(25) = 1.1, *p* = 0.28, d = 0.3) or ejaculate (t(25) = 1.2, *p* = 0.23, d = 0.2), Figure 5A‐D). Furthermore, BNST AVP knockdown caused fewer males to ejaculate with a receptive female (*x* ^2^ (2) *p* < 0.00001, φ = 1.06, Figure 5E,F). In females, BNST AVP knockdown did not affect the number of mounts received (t(20) = 1.3, *p* = 0.19, d = 0.5), intromissions received (t(20) = 1.1, *p* = 0.27, d = 0.5), or the latency to receive mounts (t(20) = 0.2, *p* = 0.82, d = 0.1), intromissions (t(20) = 0.97, *p* = 0.35, d = 0.4), or ejaculations (t(20) = 0.3, *p* = 0.78, d = 0.1)(Figure 5A–D).

### 3.6 BNST AVP knockdown did not alter anxiety‐like behaviour in the elevated plus maze

**FIGURE 6:**
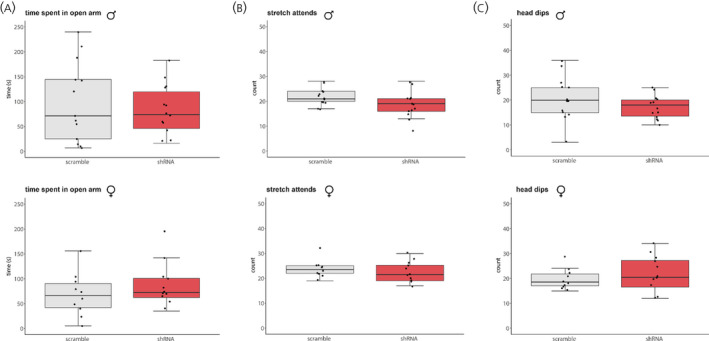
Anxiety‐like behaviour in the elevated plus maze (EPM). BNST AVP knockdown did not alter anxiety‐like behaviour. (A) *Avp*‐shRNA and scramble AAV‐injected males and females did not differ in time spent in the open arm (A), the number of stretch attend postures (B), or head dips (C). Boxplots indicate individual data points, median, first and third quartiles

We did not find sex differences in time spent in the open arms (*F*(1,45) = 1.12, *p* = 0.3, η^2^ = 0.02), the number of stretch‐attend postures (*F*(1,45) = 0.4, *p* = 0.53, η^2^ = 0.002) or head dips (*F*(1,45) = 2.05, *p* = 0.16, η^2^ = 0.02) observed in the EPM. BNST AVP knockdown did not affect any of these measures (time spent in the open arms: (*F*(1,45) = 0.01, *p* = 0.9, η^2^ = 0.001; stretch‐attend postures: (*F*(1,45) = 2.8, *p* = 0.097, η^2^ = 0.01); head dips: (*F*(1,45) = 0.1, *p* = 0.75, η^2^ = 0.001)) (Figure 6A‐C).

## 4 DISCUSSION

Previously, we found that removal of AVP‐expressing cells in the BNST of male, but not female, mice reduced investigation of same‐sex conspecifics and altered social communication, while minimally affecting female social behaviour.^19^ What was left unresolved was whether these results were due to the loss of AVP signalling from BNST cells or of other factors, such as other neuropeptides and neurotransmitters, associated with these cells. Here, we found that shRNA knockdown of AVP in BNST largely replicated the effects of BNST AVP cell ablations (e.g., on social investigation), although some effects diverged (e.g., on urine marking and copulatory behaviour), which may be related to AVP cell ablation affecting more than just AVP signalling. Additionally, BNST AVP knockdown reduced communicative behaviour (USV, tail rattles) toward same‐sex conspecifics as well as copulatory behaviour in males. Overall, this suggests that AVP from the BNST plays a more prominent role in social behaviour in males than in females and that AVP expression in the BNST drives specific aspects of social behaviour in a sexually differentiated way.

AVP produced by BNST cells may play a more impactful role in behaviour than our results indicate. As we found a 70%–80% knockdown of AVP in BNST cells, not all AVP was eliminated. In addition, since AVP cells in the MeA show similar sex differences in expression and project to overlapping areas,^11^ it may be necessary to knockdown AVP expression in both groups of cells to identify all behaviours modulated by AVP released from these cells. Furthermore, testing started three weeks after BNST AVP knockdown was initiated. As a result, additional behavioural effects of this knockdown may have disappeared due to the system adapting to chronic depletion of AVP. Such adaptation may explain the opposite effects of acute versus chronic V1aR manipulations in the lateral septum (LS) on anxiety‐like behaviour.^51^, ^52^

One of the largest effects we saw after *Avp*‐shRNA knockdown of AVP in the BNST was a reduction in male investigation of novel male conspecifics. AVP knockdown also increased the time males spent in the chamber furthest away from stimulus males, suggesting active avoidance of these males. This reduction in investigation of stimulus males was not due to deficits in overall social interest, activity level, or generalized anxiety, as investigation of female stimuli and anxiety‐like behaviours as measured in the EPM were unaltered. Also, head orientation toward the stimulus animals within the three‐chamber apparatus was not affected by AVP knockdown, indicating a specific deficit in close social investigation and not in overall attention toward the stimulus.^50^, ^53^ Additionally, we found that AVP knockdown strongly reduced male investigation of urine samples, with the largest effect seen on investigation of male urine. Together, our findings suggest that, in males, AVP produced in the BNST stimulates investigation of potentially territorial competitors and their social odors, which aligns with prior work showing that vasotocin knockdown in birds reduced social contact between males^17^ and that AVP injections into the LS, a downstream projection site for BNST AVP cells,^11^ increased male‐male interactions in rats.^54^

AVP knockdown did not affect male aggressive behaviour toward subordinate stimulus males. While these results match our previous finding that BNST AVP cell ablation affected investigative, but not aggressive, behaviour,^19^ they are somewhat unexpected, as knockdown of BNST AVP also reduced AVP‐ir fibres in the LS, a site where AVP acts on aggressive behaviour in males^18^, ^55^ and females,^56^ but see.^57^ This inconsistency may be due to differences in testing and/or procedural conditions (i.e., exposure to different types of intruders) as well as to species and strain differences.^18^, ^58^ Nevertheless, our results leave open the possibility that AVP in the LS from sources other than the BNST contribute to modulation of aggressive behaviour, as BNST AVP knockdown did not eliminate all LS AVP‐ir fibres. Therefore, AVP inputs from sources such as the medial amygdala and PVN^10^ may contribute to AVP effects within the LS on aggressive behaviour.

Although BNST AVP knockdown did not change offensive attack behaviour, it still modestly reduced male signalling behaviour in potentially antagonistic settings. Specifically, AVP knockdown reduced tail rattling, a known component of aggressive behaviour by dominant males,^59^ and USVs in the presence of other males. However, knockdown did not reduce USVs produced in the presence of receptive females. Typically, males vocalize more than females^60^ and primarily direct their vocalizations toward females^61^; however, males do produce USVs in male‐male territorial contexts.^61^, ^62^ Therefore, our results suggest that BNST AVP is involved in some aspects of male offensive/territorial signalling (e.g., USVs and tail rattling).

In agreement with the effects of BNST AVP cell ablation,^19^, ^20^ we observed no changes in anxiety‐like behaviour following BNST AVP knockdown. This was somewhat unexpected, as central AVP has been implicated in the modulation of anxiety.^63^, ^64^, ^65^ Moreover, pharmacological studies suggest that AVP in the LS, a target of BNST AVP cells in mice, may modulate anxiety.^51^, ^57^, ^66^ However, the present results are consistent with observations that electrolytic lesions of the BNST had no effect on anxiety‐like behaviour in the EPM in rats.^67^ Therefore, AVP derived from other sources may modulate anxiety action when released in the septum. We do note, however, that ablation of AVP cell groups in the PVN in males, or in the suprachiasmatic nucleus (SCN) in both sexes, increased anxiety‐like behaviour in the EPM.^68^, ^69^ Consequently, the LS may require AVP signals from both BNST and MeA to increase anxiety‐like behaviours, while other sources of AVP may work to inhibit it.

While BNST AVP knockdown and BNST AVP cell ablation^19^ have similar effects or lack thereof on male‐male social investigation, aggression, and anxiety, AVP knockdown did not replicate other behavioural effects of BNST AVP cell deletion. For example, BNST AVP cell ablation in males increased urine marking toward females, whereas BNST AVP knockdown did not.^19^ It may be that BNST AVP cell ablations are more effective in reducing AVP levels than AVP knockdown. However, the percentage of reduction of AVP‐ir cells in the BNST was high in both studies (shRNA: ~70%–80% average; cell ablations: ~90%+ average). The most parsimonious explanation for this is that AVP cell ablation may have removed more signalling molecules than just AVP, each of which may have effects on behaviour.

Our results demonstrate a distinct, sexually differentiated role of BNST AVP in male copulatory behaviour. Knockdown of AVP within the BNST reduced intromissions and ejaculations in males, but did not alter copulatory behaviour in females. These results are broadly consistent with the observation that BNST and MeA AVP cells are active during male copulatory behaviour.^70^, ^71^ As we did not observe changes in precopulatory (male investigation, USVs, urine marking) or mounting behaviours toward receptive females after AVP knockdown, the effects on copulatory behaviour are more likely to be due to deficits in bridging the appetitive and consummatory phases of sexual behaviour, rather than to changes in sexual motivation.^72^ These results do not match the effects of ablating BNST AVP cells, which reduced female, but not male, copulatory behaviour.^19^ One possible explanation for this discrepancy is that AVP ablation left the expression of galanin, a neuropeptide colocalized with AVP in the BNST^73^ intact in the present study. This may have increased overall inhibitory signalling to targets of BNST AVP cells, as galanin promotes neuronal inhibition^74^ whereas AVP promotes excitation.^65^ Indeed, ICV injections of galanin strongly inhibited male copulatory behaviour in rats,^75^ and galanin has been shown to block AVP‐induced flank marking in golden hamsters.^76^ Future studies may help unravel the physiological and behavioural significance of coexpression of AVP and other signalling molecules.

## 5 CONCLUSION

In summary, our results indicate that sexually dimorphic AVP expression within the BNST contributes to sex differences in social behaviour. More specifically, BNST AVP knockdown in male, but not female, mice reduced investigation and communicative behaviours directed toward same‐sex conspecifics as well as male sexual behaviour, without changing offensive attacks, anxiety‐related behaviours, and overall activity. These results match those of other studies that have shown a sex‐specific role of AVP in behavior. For example, AVP and its antagonists have different effects on aggressive play behaviour in rats,^77^ territorial aggression in hamsters,^78^, ^79^, ^80^ and social communication in humans.^81^, ^82^, ^83^ Together, these studies point toward a sexually differentiated role of AVP in vertebrate social behaviour. By ablating specific AVP cell groups in the BNST, PVN, SCN,^19^, ^68^, ^69^ or knocking down AVP specifically in the BNST (present results), we have started addressing directly the question as to which AVP cell groups contribute to sex differences in social behaviour and its regulation.

## CONFLICT OF INTEREST

The authors report no conflict of interest.

## AUTHOR CONTRIBUTIONS

**Nicole Rigney:** Conceptualization; Data curation; Formal analysis; Funding acquisition; Investigation; Methodology; Project administration; Supervision; Validation; Visualization; Writing – original draft; Writing – review and editing. **Adam Zbib:** Data curation; Formal analysis; Writing – original draft. **Geert J. de Vries:** Conceptualization; Funding acquisition; Supervision; Writing – original draft; Writing – review and editing. **Aras Petrulis:** Conceptualization; Funding acquisition; Supervision; Writing – original draft; Writing – review and editing.

### PEER REVIEW

The peer review history for this article is available at https://publons.com/publon/10.1111/jne.13083.

## Supporting information

Fig S1‐S6

## Data Availability

The data that support the findings of this study are available from the corresponding author upon reasonable request.
