# Supplementary material for: Knockdown of sexually differentiated vasopressin expression in the bed nucleus of the stria terminalis reduces social and sexual behaviour in male, but not female, mice
Source: J Neuroendocrinol. 2022 Jan 2;34(9):e13083. doi: 10.1111/jne.13083 (PMC9213575; doi:10.1111/jne.13083)
Supplement: Supplementary file 1 — Fig S1‐S6 [file JNE-34-e13083-s001.docx]

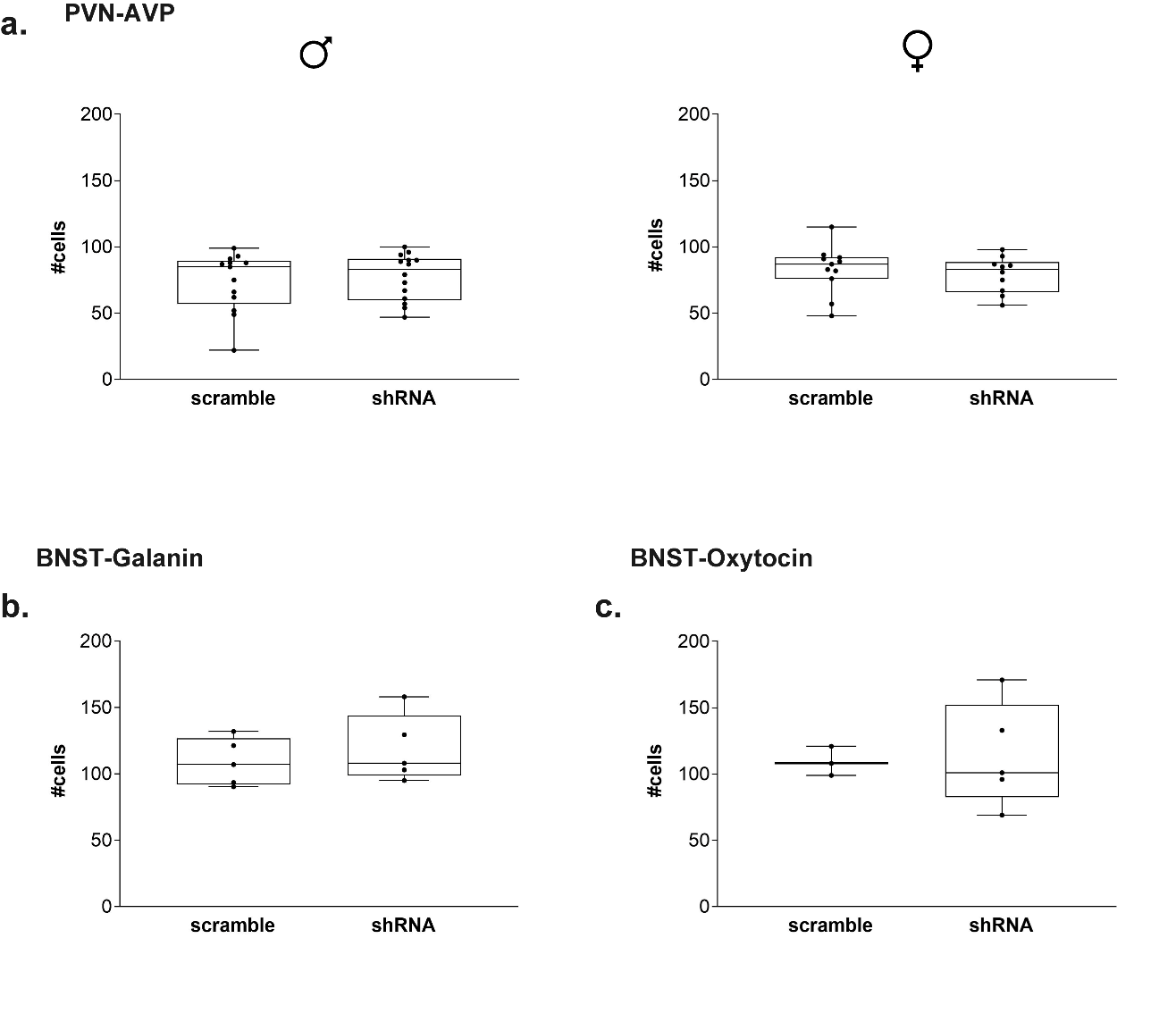


**Supplementary Fig. 1.** Histology. **(a)** Boxplot of AVP-ir cell number within the paraventricular nucleus of the hypothalamus (PVN). There was no difference in AVP-ir cells within the PVN in both *Avp-*shRNA-injected male and female mice compared to scramble shRNA- injected control mice (males: p = 0.6; females: p = 0.58). *Avp*-shRNA (n = 14) and scramble (n = 13) injected males and *AVP*-shRNA (n = 10) and scramble (n = 11) injected females. **(b)** Boxplot of galanin-ir cell number within the BNST. There were no differences in the number of galanin-ir cells in *AVP* shRNA-injected male mice compared to scramble shRNA-injected control mice, p = 0.5. *Avp*-shRNA (n = 5) and scramble (n = 5) injected males. **(c)** Boxplot of oxytocin (OT)-ir cell number within the BNST. There were no differences in the number of OT-ir cells in shRNA injected male mice compared to scramble injected control mice, p = 0.85. *Avp*-shRNA (n = 5) and scramble (n = 3) injected males. Boxplots indicate individual data points, median, first, and third quartiles.


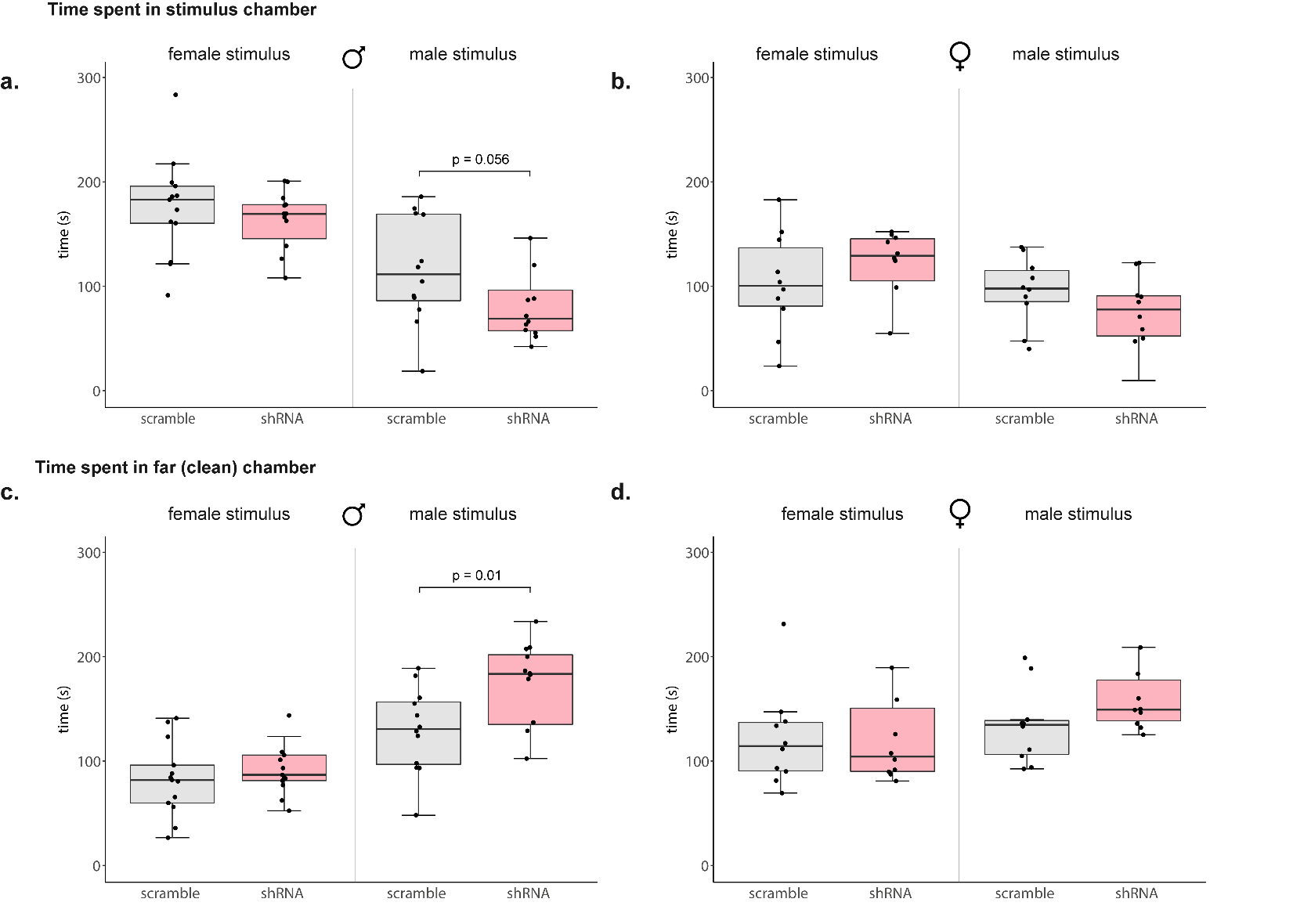


**Supplementary Fig. 2** Time spent in the stimulus or clean chamber of the three-chamber apparatus. **(a)** There was a trend toward *Avp*-shRNA-injected males to spend less time in the chamber containing a male stimulus compared to scramble shRNA control (p = 0.056), but there was no difference between *Avp*-shRNA and scramble shRNA-injected females **(b)**. **(c)** *Avp*-shRNA-injected males spent more time in the chamber furthest away from the male stimulus (p = 0.01), and again, **(d)** there was no difference between *Avp*-shRNA and scramble shRNA-injected females. Boxplots indicate individual data points, median, first, and third quartiles.


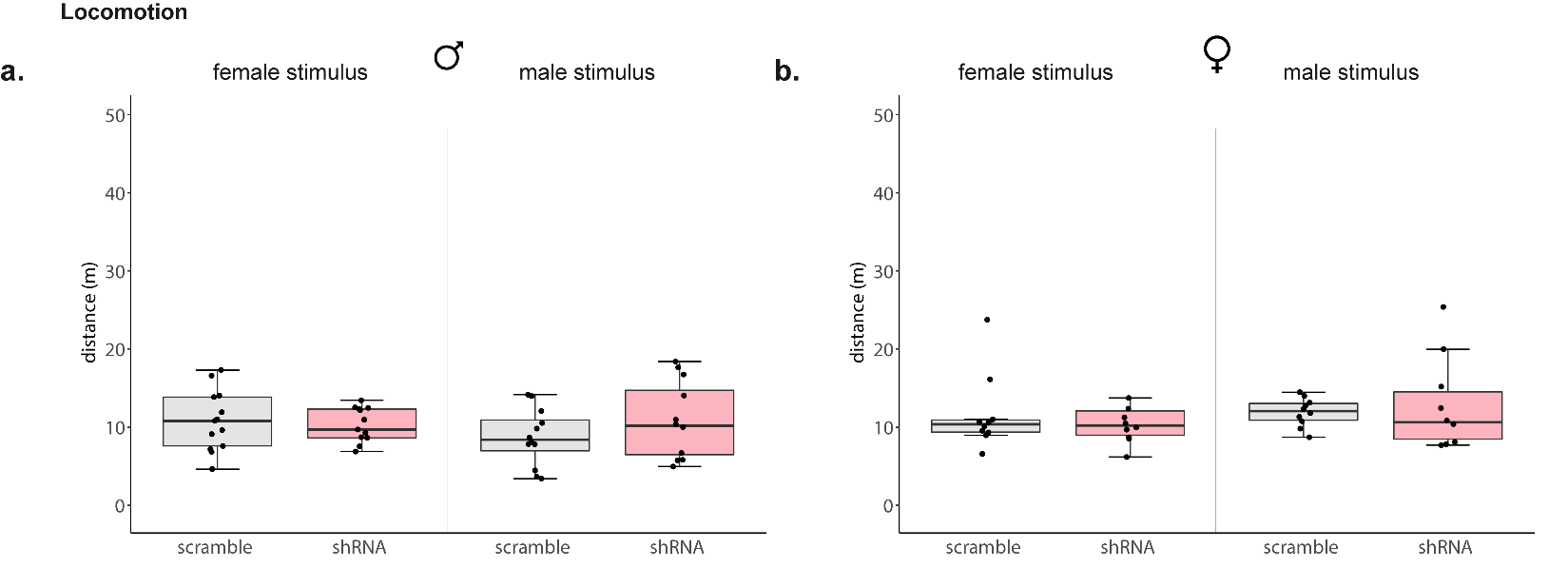


**Supplementary Fig. 3** Locomotion. **(a, b)** Scramble shRNA control and *Avp*-shRNA subjects did not differ in the distance traveled within the three-chamber apparatus. Boxplots indicate individual data points, median, first, and third quartiles.


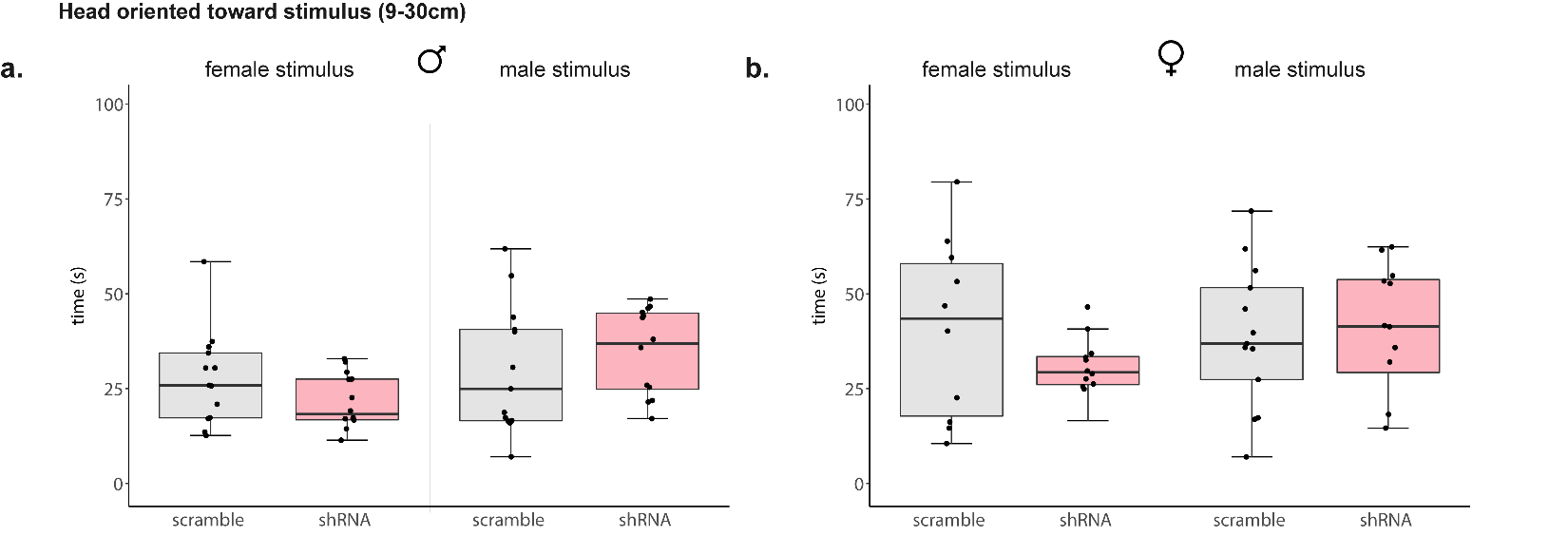


**Supplementary Fig. 4** Head orientation toward male and female stimuli. **(a, b)** Scramble control and *Avp*-shRNA subjects did not differ in the time spent oriented toward stimuli at a 9-30cm distance within the three-chamber apparatus. Boxplots indicate individual data points, median, first, and third quartiles.


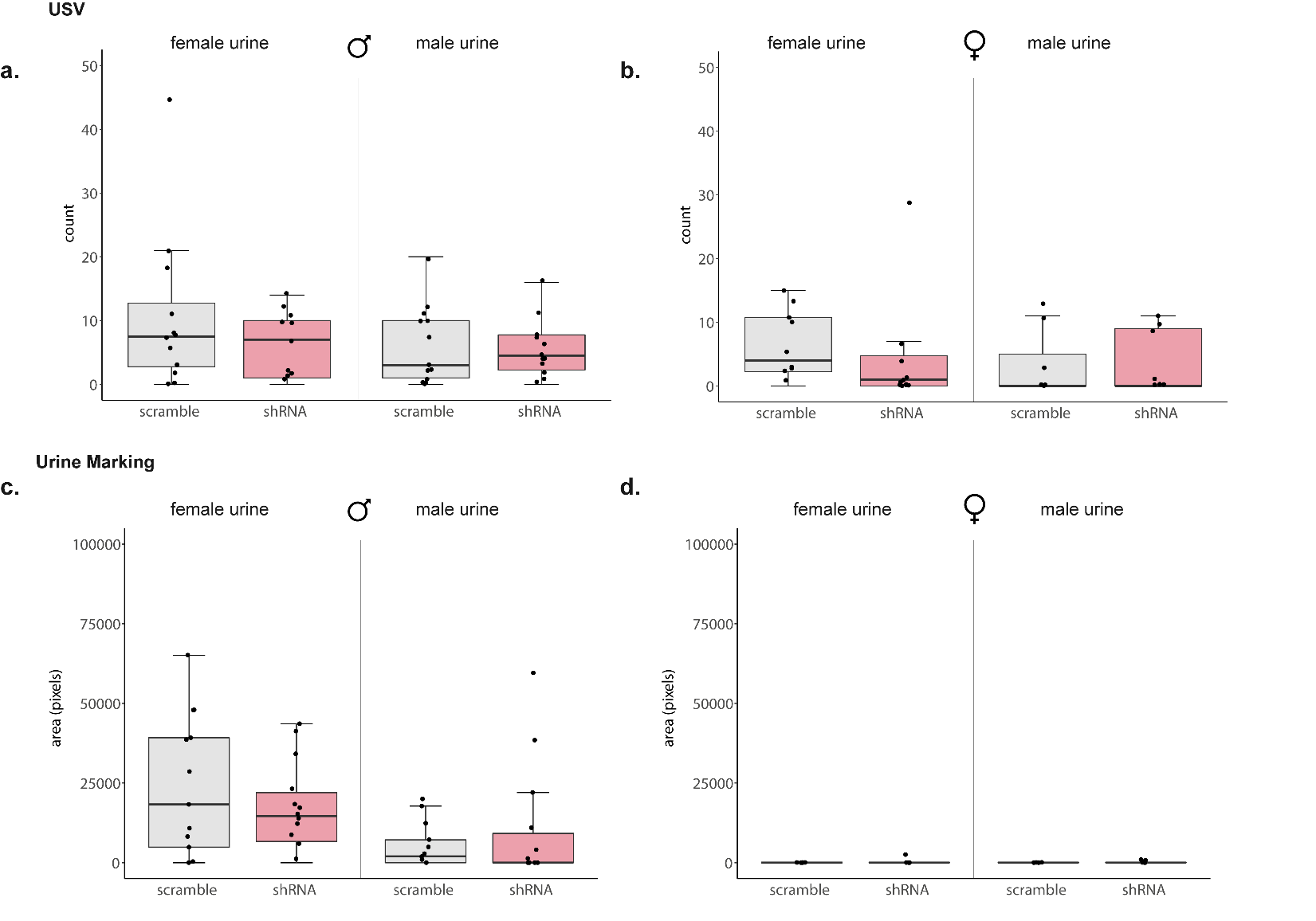


**Supplementary Fig. 5** Ultrasonic vocalizations (USV) and urine marking toward male and female urine stimuli within the three-chamber apparatus. **(a-b)** *Avp*-shRNA and scramble shRNA-injected males **(a)** and females **(b)** did not differ in USVs produced toward urine stimuli. **(c-d)** *Avp*-shRNA and scramble shRNA-injected males **(c)** and females **(d)** did not differ in urine marking toward urine stimuli. Boxplots indicate individual data points, median, first and third quartiles.
